# (*E*)-1-[4-(Hex­yloxy)phen­yl]-3-(2-hy­droxy­phen­yl)prop-2-en-1-one

**DOI:** 10.1107/S1600536812038007

**Published:** 2012-09-08

**Authors:** Siti Muhaini Haris Fadzillah, Zainab Ngaini, Hasnain Hussain, Ibrahim Abdul Razak, Safra Izuani Jama Asik

**Affiliations:** a Department of Chemistry, Faculty of Resource Science and Technology, Universiti Malaysia Sarawak, 94300 Kota Samarahan, Sarawak, Malaysia; b Department of Molecular Biology, Faculty of Resource Science and Technology, Universiti Malaysia Sarawak, 94300 Kota Samarahan, Sarawak, Malaysia; c School of Physics, Universiti Sains Malaysia, 11800 USM, Penang, Malaysia

## Abstract

In the title compound, C_21_H_24_O_3_, the enone moiety adopts an *s-cis* conformation and the dihedral angle between the benzene rings is 12.89 (6)°. The hex­yloxy tail adopts an extended conformation. In the crystal, inversion dimers are linked by pairs of O—H⋯O hydrogen bonds and pairs of C—H⋯O inter­actions, forming two *R*_2_^2^(7) and one *R*_2_^2^(10) loops. The dimers are then arranged into sheets lying parallel to (201) and weak C—H⋯π inter­actions consolidate the packing.

## Related literature

For a related structure and background to the biological properties of chalcones, see: Ngaini *et al.* (2011 ▶). For related structures, see: Razak *et al.* (2009 ▶); Ngaini *et al.* (2010 ▶). For graph-set theory, see: Bernstein *et al.* (1995 ▶). For the stability of the temperature controller used in the data collection, see: Cosier & Glazer (1986) ▶.
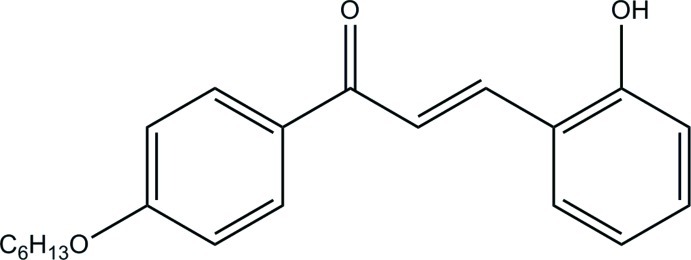


## Experimental

### 

#### Crystal data

C_21_H_24_O_3_
*M* *_r_* = 324.40
Triclinic, 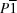

*a* = 7.485 (2) Å
*b* = 10.834 (3) Å
*c* = 11.673 (3) Å
α = 73.858 (5)°
β = 77.961 (6)°
γ = 76.941 (6)°
*V* = 874.9 (4) Å^3^
*Z* = 2
Mo *K*α radiation
μ = 0.08 mm^−1^
*T* = 100 K
0.47 × 0.14 × 0.12 mm

#### Data collection

Bruker APEX DUO CCD diffractometer
Absorption correction: multi-scan (*SADABS*; Bruker, 2009 ▶) *T*_min_ = 0.963, *T*_max_ = 0.991
17565 measured reflections
4576 independent reflections
3781 reflections with *I* > 2σ(*I*)
*R*_int_ = 0.026

#### Refinement

*R*[*F* ^2^ > 2σ(*F* ^2^)] = 0.041
*wR*(*F* ^2^) = 0.133
*S* = 1.02
4576 reflections
222 parameters
H atoms treated by a mixture of independent and constrained refinement
Δρ_max_ = 0.39 e Å^−3^
Δρ_min_ = −0.23 e Å^−3^

### 

Data collection: *APEX2* (Bruker, 2009 ▶); cell refinement: *SAINT* (Bruker, 2009 ▶); data reduction: *SAINT*; program(s) used to solve structure: *SHELXTL* (Sheldrick, 2008 ▶); program(s) used to refine structure: *SHELXTL*; molecular graphics: *SHELXTL*; software used to prepare material for publication: *SHELXTL* and *PLATON* (Spek, 2009 ▶).

## Supplementary Material

Crystal structure: contains datablock(s) global, I. DOI: 10.1107/S1600536812038007/hb6948sup1.cif

Structure factors: contains datablock(s) I. DOI: 10.1107/S1600536812038007/hb6948Isup2.hkl

Supplementary material file. DOI: 10.1107/S1600536812038007/hb6948Isup3.cml

Additional supplementary materials:  crystallographic information; 3D view; checkCIF report

## Figures and Tables

**Table 1:** Hydrogen-bond geometry (Å, °) *Cg*1 is the centroid of the C1–C6 ring.

*D*—H⋯*A*	*D*—H	H⋯*A*	*D*⋯*A*	*D*—H⋯*A*
O2—H1*O*2⋯O1^i^	0.94 (2)	1.78 (2)	2.6862 (15)	163.3 (19)
C7—H7*A*⋯O2^i^	0.93	2.37	3.2526 (18)	158
C16—H16*A*⋯*Cg*1^ii^	0.97	2.91	3.6117 (16)	130

Symmetry codes: (i) 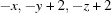; (ii) 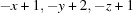.

**Fig. 1.**
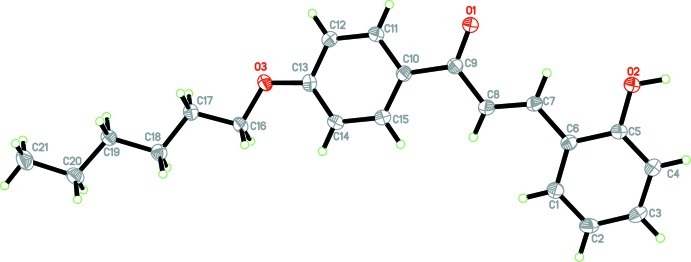
The structure of the title compound, showing 50% probability displacement ellipsoids.

**Fig. 2.**
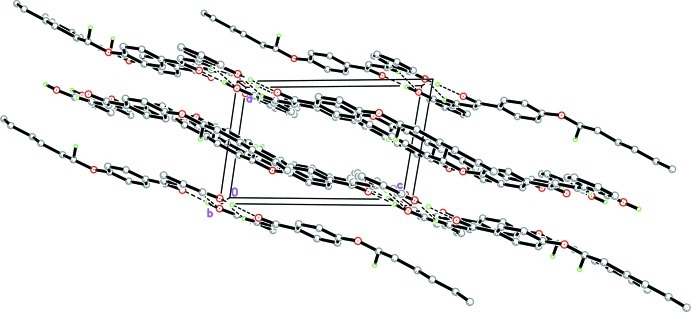
The crystal packing, viewed along the a-axis, showing the molecules in pairs, arranged into sheets parallel to (201) plane. Hydrogen bonds are shown as dashed lines.

## supplementary crystallographic information

### Comment

As part of our ongoing studies of the biological activities of chalcone derivatives (Ngaini *et al.*, 2011), the title compound has been synthesised and tested against *E. coli* ATCC 8739 and showed anti-microbial activity. We now describe its crystal structure.

In the title of chalcone derivative, Fig. 1, the conformation of the enone (O1/C7–C8) moiety is *s-cis* with the C7–C8–C9–o1 torsion angle being 0.89 (18)°. The least-square plane through enone moeity make dihedral angles of 7.57 (7)° and 8.18 (7)° with (C10–C15 and C1–C6) benzene rings, respectively. The dihedral angle between the two benzene rings is 12.89 (6)°.

The widening of C9–C10–C15, C6–C7–C8 and C1–C6–C7 angles to 123.98 (10)°, 126.08 (10)° and 123.26 (10) respectively, are the consequences of the short contact between H15A and H8A (2.075 Å) as well as H8A and H1A (2.214 Å). Likewise, the slight opening of O3–C13–C14 to 125.30 (10)° is the result of the strain induced by close H14A···H16A (2.253 Å) interatomic contact. Similar features were also observed in closely related structures (Razak *et al.*, 2009; Ngaini *et al.*, 2010; Ngaini *et al.*, 2011).

The zigzag alkoxyl tail is assumed as a *trans* conformation. The torsion angle C16–O3–C13–C14 of 2.51 (16)° implies that the alkoxyl tail is roughly co-planar with the attached benzene ring. However, it is actually twisted away from planarity as shown by the torsion angle of 166.42 (9)° for C13–O3–C16–C17. The twist about C17–C18 bond is indicated by C16–C17–C18–C19 torsion angle of 173.51 (9)°.

In the crystal packing of (Fig. 2), the molecules are connected by intermolecular interactions O2—H1O2···O1 and C7—H7A···O2 hydrogen bonds to form two *R*^2^_2_(7) and one *R*^2^_2_(10) rings motif which linked the molecules into pairs which are then arranged into sheets parallel to (201) plane. Furthermore, the crystal packing features weak C—H···π interactions (Table 1) with the distance of 3.6117 (16) Å.

### Experimental

A mixture of 2-hydroxybenzaldehyde (1.46 ml, 12 mmol), and 4-hexyloxyacetophenone (2.64 g, 12 mmol) and KOH (2.42 g, 43 mmol) in methanol (50 ml) was heated at reflux for 24 h. The reaction was cooled to room temperature and acidified with cold diluted HCl (2N). The resulting precipitate was filtered, washed and dried. After redissolving in hexane-ethanol (7:1) followed by few days of slow evaporation, yellow blocks were collected.

### Refinement

The O-bound H atom was located in a difference Fourier map and refined freely with O–H = 0.94 (2) Å. The remaining H atoms were placed in calculated positions with C–H = 0.93–0.97 Å. The *U*_iso_ values were constrained to be 1.5*U*_eq_ (methyl-H atom) and 1.2*U*_eq_ (other H atoms). The rotating model group was applied for the methyl group.

### Crystal data

**Table:**

C_21_H_24_O_3_	*Z* = 2
*M**_r_* = 324.40	*F*(000) = 348
Triclinic, *P*1	*D*_x_ = 1.231 Mg m^−^^3^
Hall symbol: -P 1	Mo *K*α radiation, λ = 0.71073 Å
*a* = 7.485 (2) Å	Cell parameters from 6758 reflections
*b* = 10.834 (3) Å	θ = 2.4–31.7°
*c* = 11.673 (3) Å	µ = 0.08 mm^−^^1^
α = 73.858 (5)°	*T* = 100 K
β = 77.961 (6)°	Block, yellow
γ = 76.941 (6)°	0.47 × 0.14 × 0.12 mm
*V* = 874.9 (4) Å^3^	

### Data collection

**Table:**

Bruker APEX DUO CCD diffractometer	4576 independent reflections
Radiation source: fine-focus sealed tube	3781 reflections with *I* > 2σ(*I*)
Graphite monochromator	*R*_int_ = 0.026
φ and ω scans	θ_max_ = 29.0°, θ_min_ = 1.8°
Absorption correction: multi-scan (*SADABS*; Bruker, 2009)	*h* = −10→10
*T*_min_ = 0.963, *T*_max_ = 0.991	*k* = −14→14
17565 measured reflections	*l* = −15→15

### Refinement

**Table:**

Refinement on *F*^2^	Primary atom site location: structure-invariant direct methods
Least-squares matrix: full	Secondary atom site location: difference Fourier map
*R*[*F*^2^ > 2σ(*F*^2^)] = 0.041	Hydrogen site location: inferred from neighbouring sites
*wR*(*F*^2^) = 0.133	H atoms treated by a mixture of independent and constrained refinement
*S* = 1.02	*w* = 1/[σ^2^(*F*_o_^2^) + (0.077*P*)^2^ + 0.2082*P*] where *P* = (*F*_o_^2^ + 2*F*_c_^2^)/3
4576 reflections	(Δ/σ)_max_ < 0.001
222 parameters	Δρ_max_ = 0.39 e Å^−^^3^
0 restraints	Δρ_min_ = −0.23 e Å^−^^3^

### Special details

**Table:**

Experimental. The crystal was placed in the cold stream of an Oxford Cryosystems Cobra open-flow nitrogen cryostat (Cosier & Glazer, 1986) operating at 100.0 (1) K.
Geometry. All esds (except the esd in the dihedral angle between two l.s. planes) are estimated using the full covariance matrix. The cell esds are taken into account individually in the estimation of esds in distances, angles and torsion angles; correlations between esds in cell parameters are only used when they are defined by crystal symmetry. An approximate (isotropic) treatment of cell esds is used for estimating esds involving l.s. planes.
Refinement. Refinement of F^2^ against ALL reflections. The weighted R-factor wR and goodness of fit S are based on F^2^, conventional R-factors R are based on F, with F set to zero for negative F^2^. The threshold expression of F^2^ > 2sigma(F^2^) is used only for calculating R-factors(gt) etc. and is not relevant to the choice of reflections for refinement. R-factors based on F^2^ are statistically about twice as large as those based on F, and R- factors based on ALL data will be even larger.

### Fractional atomic coordinates and isotropic or equivalent isotropic displacement parameters (Å^2^)

**Table:**

	*x*	*y*	*z*	*U*_iso_*/*U*_eq_	
O1	0.10199 (12)	0.82681 (7)	0.77465 (7)	0.02537 (19)	
O2	0.05000 (12)	1.13926 (7)	1.00412 (7)	0.02580 (19)	
O3	0.26974 (11)	0.74559 (8)	0.24879 (7)	0.02478 (19)	
C1	0.23994 (15)	1.29725 (10)	0.70042 (10)	0.0229 (2)	
H1A	0.2848	1.2773	0.6260	0.027*	
C2	0.24424 (16)	1.41983 (11)	0.71304 (11)	0.0261 (2)	
H2A	0.2922	1.4812	0.6477	0.031*	
C3	0.17661 (16)	1.45074 (10)	0.82374 (11)	0.0256 (2)	
H3A	0.1763	1.5338	0.8318	0.031*	
C4	0.10963 (15)	1.35851 (10)	0.92229 (10)	0.0232 (2)	
H4A	0.0655	1.3794	0.9964	0.028*	
C5	0.10858 (14)	1.23422 (10)	0.91002 (10)	0.0198 (2)	
C6	0.16962 (14)	1.20282 (10)	0.79729 (10)	0.0194 (2)	
C7	0.15502 (14)	1.07449 (10)	0.78689 (10)	0.0205 (2)	
H7A	0.1211	1.0145	0.8581	0.025*	
C8	0.18600 (15)	1.03496 (10)	0.68408 (10)	0.0226 (2)	
H8A	0.2266	1.0912	0.6119	0.027*	
C9	0.15789 (14)	0.90539 (10)	0.68181 (10)	0.0201 (2)	
C10	0.19366 (14)	0.86986 (10)	0.56431 (10)	0.0199 (2)	
C11	0.14263 (15)	0.75378 (10)	0.56113 (10)	0.0223 (2)	
H11A	0.0896	0.7018	0.6320	0.027*	
C12	0.17005 (15)	0.71606 (11)	0.45458 (10)	0.0239 (2)	
H12A	0.1349	0.6392	0.4541	0.029*	
C13	0.25034 (14)	0.79245 (10)	0.34708 (10)	0.0213 (2)	
C14	0.30205 (15)	0.90824 (11)	0.34783 (10)	0.0235 (2)	
H14A	0.3550	0.9600	0.2767	0.028*	
C15	0.27332 (15)	0.94525 (10)	0.45625 (10)	0.0232 (2)	
H15A	0.3082	1.0222	0.4567	0.028*	
C16	0.35636 (15)	0.81399 (11)	0.13354 (10)	0.0226 (2)	
H16A	0.4699	0.8376	0.1415	0.027*	
H16B	0.2734	0.8929	0.1006	0.027*	
C17	0.39770 (15)	0.72005 (10)	0.05308 (10)	0.0225 (2)	
H17A	0.2820	0.6985	0.0464	0.027*	
H17B	0.4742	0.6400	0.0907	0.027*	
C18	0.49635 (15)	0.77343 (11)	−0.07282 (10)	0.0227 (2)	
H18A	0.6055	0.8039	−0.0667	0.027*	
H18B	0.4144	0.8473	−0.1147	0.027*	
C19	0.55427 (16)	0.66941 (11)	−0.14528 (10)	0.0243 (2)	
H19A	0.6362	0.5961	−0.1027	0.029*	
H19B	0.4446	0.6381	−0.1491	0.029*	
C20	0.65178 (16)	0.71628 (12)	−0.27296 (10)	0.0275 (2)	
H20A	0.7597	0.7501	−0.2699	0.033*	
H20B	0.5685	0.7871	−0.3171	0.033*	
C21	0.7131 (2)	0.60782 (14)	−0.34014 (12)	0.0375 (3)	
H21A	0.7817	0.6400	−0.4180	0.056*	
H21B	0.6058	0.5795	−0.3500	0.056*	
H21C	0.7904	0.5356	−0.2947	0.056*	
H1O2	0.001 (3)	1.1676 (18)	1.0758 (18)	0.058 (5)*	

### Atomic displacement parameters (Å^2^)

**Table:**

	*U*^11^	*U*^22^	*U*^33^	*U*^12^	*U*^13^	*U*^23^
O1	0.0357 (4)	0.0204 (4)	0.0196 (4)	−0.0082 (3)	−0.0005 (3)	−0.0046 (3)
O2	0.0370 (4)	0.0201 (4)	0.0197 (4)	−0.0094 (3)	0.0018 (3)	−0.0052 (3)
O3	0.0294 (4)	0.0283 (4)	0.0181 (4)	−0.0101 (3)	0.0017 (3)	−0.0081 (3)
C1	0.0254 (5)	0.0209 (5)	0.0214 (5)	−0.0045 (4)	−0.0036 (4)	−0.0033 (4)
C2	0.0300 (5)	0.0200 (5)	0.0266 (6)	−0.0080 (4)	−0.0036 (4)	−0.0009 (4)
C3	0.0291 (5)	0.0180 (5)	0.0315 (6)	−0.0062 (4)	−0.0065 (4)	−0.0058 (4)
C4	0.0258 (5)	0.0209 (5)	0.0252 (6)	−0.0040 (4)	−0.0051 (4)	−0.0084 (4)
C5	0.0207 (5)	0.0183 (5)	0.0203 (5)	−0.0048 (4)	−0.0036 (4)	−0.0032 (4)
C6	0.0200 (4)	0.0173 (4)	0.0211 (5)	−0.0030 (4)	−0.0041 (4)	−0.0043 (4)
C7	0.0226 (5)	0.0164 (4)	0.0220 (5)	−0.0038 (4)	−0.0032 (4)	−0.0038 (4)
C8	0.0285 (5)	0.0184 (5)	0.0205 (5)	−0.0052 (4)	−0.0037 (4)	−0.0035 (4)
C9	0.0214 (5)	0.0184 (5)	0.0198 (5)	−0.0023 (4)	−0.0028 (4)	−0.0048 (4)
C10	0.0214 (5)	0.0179 (5)	0.0197 (5)	−0.0018 (4)	−0.0027 (4)	−0.0051 (4)
C11	0.0267 (5)	0.0193 (5)	0.0193 (5)	−0.0054 (4)	−0.0006 (4)	−0.0035 (4)
C12	0.0274 (5)	0.0218 (5)	0.0239 (6)	−0.0078 (4)	−0.0007 (4)	−0.0074 (4)
C13	0.0209 (5)	0.0234 (5)	0.0197 (5)	−0.0022 (4)	−0.0028 (4)	−0.0073 (4)
C14	0.0279 (5)	0.0222 (5)	0.0194 (5)	−0.0076 (4)	0.0000 (4)	−0.0036 (4)
C15	0.0276 (5)	0.0201 (5)	0.0220 (5)	−0.0073 (4)	−0.0013 (4)	−0.0051 (4)
C16	0.0236 (5)	0.0263 (5)	0.0168 (5)	−0.0056 (4)	−0.0002 (4)	−0.0047 (4)
C17	0.0227 (5)	0.0238 (5)	0.0205 (5)	−0.0028 (4)	−0.0022 (4)	−0.0064 (4)
C18	0.0241 (5)	0.0233 (5)	0.0198 (5)	−0.0029 (4)	−0.0039 (4)	−0.0044 (4)
C19	0.0287 (5)	0.0251 (5)	0.0192 (5)	−0.0080 (4)	−0.0010 (4)	−0.0052 (4)
C20	0.0303 (6)	0.0317 (6)	0.0202 (5)	−0.0099 (5)	−0.0004 (4)	−0.0049 (4)
C21	0.0431 (7)	0.0501 (8)	0.0255 (6)	−0.0195 (6)	0.0053 (5)	−0.0180 (6)

### Geometric parameters (Å, º)

**Table:**

O1—C9	1.2382 (13)	C12—C13	1.3976 (15)
O2—C5	1.3534 (13)	C12—H12A	0.9300
O2—H1O2	0.94 (2)	C13—C14	1.3967 (15)
O3—C13	1.3477 (13)	C14—C15	1.3922 (15)
O3—C16	1.4416 (13)	C14—H14A	0.9300
C1—C2	1.3844 (15)	C15—H15A	0.9300
C1—C6	1.3978 (15)	C16—C17	1.5106 (15)
C1—H1A	0.9300	C16—H16A	0.9700
C2—C3	1.3891 (17)	C16—H16B	0.9700
C2—H2A	0.9300	C17—C18	1.5189 (16)
C3—C4	1.3856 (16)	C17—H17A	0.9700
C3—H3A	0.9300	C17—H17B	0.9700
C4—C5	1.3951 (14)	C18—C19	1.5263 (15)
C4—H4A	0.9300	C18—H18A	0.9700
C5—C6	1.4086 (15)	C18—H18B	0.9700
C6—C7	1.4587 (14)	C19—C20	1.5181 (16)
C7—C8	1.3424 (15)	C19—H19A	0.9700
C7—H7A	0.9300	C19—H19B	0.9700
C8—C9	1.4738 (14)	C20—C21	1.5247 (17)
C8—H8A	0.9300	C20—H20A	0.9700
C9—C10	1.4815 (15)	C20—H20B	0.9700
C10—C15	1.3953 (15)	C21—H21A	0.9600
C10—C11	1.4064 (14)	C21—H21B	0.9600
C11—C12	1.3763 (15)	C21—H21C	0.9600
C11—H11A	0.9300		
			
C5—O2—H1O2	113.6 (11)	C15—C14—H14A	120.4
C13—O3—C16	119.91 (8)	C13—C14—H14A	120.4
C2—C1—C6	121.38 (11)	C14—C15—C10	121.74 (10)
C2—C1—H1A	119.3	C14—C15—H15A	119.1
C6—C1—H1A	119.3	C10—C15—H15A	119.1
C1—C2—C3	119.72 (10)	O3—C16—C17	105.61 (9)
C1—C2—H2A	120.1	O3—C16—H16A	110.6
C3—C2—H2A	120.1	C17—C16—H16A	110.6
C4—C3—C2	120.39 (10)	O3—C16—H16B	110.6
C4—C3—H3A	119.8	C17—C16—H16B	110.6
C2—C3—H3A	119.8	H16A—C16—H16B	108.7
C3—C4—C5	119.82 (10)	C16—C17—C18	113.53 (9)
C3—C4—H4A	120.1	C16—C17—H17A	108.9
C5—C4—H4A	120.1	C18—C17—H17A	108.9
O2—C5—C4	122.26 (10)	C16—C17—H17B	108.9
O2—C5—C6	117.18 (9)	C18—C17—H17B	108.9
C4—C5—C6	120.56 (10)	H17A—C17—H17B	107.7
C1—C6—C5	118.05 (9)	C17—C18—C19	111.28 (9)
C1—C6—C7	123.26 (10)	C17—C18—H18A	109.4
C5—C6—C7	118.68 (9)	C19—C18—H18A	109.4
C8—C7—C6	126.09 (10)	C17—C18—H18B	109.4
C8—C7—H7A	117.0	C19—C18—H18B	109.4
C6—C7—H7A	117.0	H18A—C18—H18B	108.0
C7—C8—C9	122.19 (10)	C20—C19—C18	114.28 (9)
C7—C8—H8A	118.9	C20—C19—H19A	108.7
C9—C8—H8A	118.9	C18—C19—H19A	108.7
O1—C9—C8	121.95 (10)	C20—C19—H19B	108.7
O1—C9—C10	119.27 (9)	C18—C19—H19B	108.7
C8—C9—C10	118.76 (9)	H19A—C19—H19B	107.6
C15—C10—C11	117.94 (10)	C19—C20—C21	112.35 (10)
C15—C10—C9	123.98 (9)	C19—C20—H20A	109.1
C11—C10—C9	118.07 (9)	C21—C20—H20A	109.1
C12—C11—C10	120.94 (10)	C19—C20—H20B	109.1
C12—C11—H11A	119.5	C21—C20—H20B	109.1
C10—C11—H11A	119.5	H20A—C20—H20B	107.9
C11—C12—C13	120.47 (10)	C20—C21—H21A	109.5
C11—C12—H12A	119.8	C20—C21—H21B	109.5
C13—C12—H12A	119.8	H21A—C21—H21B	109.5
O3—C13—C14	125.30 (10)	C20—C21—H21C	109.5
O3—C13—C12	115.01 (9)	H21A—C21—H21C	109.5
C14—C13—C12	119.69 (10)	H21B—C21—H21C	109.5
C15—C14—C13	119.22 (10)		
			
C6—C1—C2—C3	−0.37 (17)	C8—C9—C10—C11	−171.57 (9)
C1—C2—C3—C4	1.75 (17)	C15—C10—C11—C12	−0.18 (16)
C2—C3—C4—C5	−0.56 (16)	C9—C10—C11—C12	179.21 (9)
C3—C4—C5—O2	177.95 (9)	C10—C11—C12—C13	0.31 (17)
C3—C4—C5—C6	−2.02 (16)	C16—O3—C13—C14	2.51 (16)
C2—C1—C6—C5	−2.13 (16)	C16—O3—C13—C12	−178.00 (9)
C2—C1—C6—C7	177.29 (10)	C11—C12—C13—O3	−179.89 (9)
O2—C5—C6—C1	−176.65 (9)	C11—C12—C13—C14	−0.37 (16)
C4—C5—C6—C1	3.32 (15)	O3—C13—C14—C15	179.78 (10)
O2—C5—C6—C7	3.90 (14)	C12—C13—C14—C15	0.31 (16)
C4—C5—C6—C7	−176.13 (9)	C13—C14—C15—C10	−0.19 (17)
C1—C6—C7—C8	−7.76 (17)	C11—C10—C15—C14	0.12 (16)
C5—C6—C7—C8	171.66 (10)	C9—C10—C15—C14	−179.23 (10)
C6—C7—C8—C9	−176.61 (9)	C13—O3—C16—C17	166.42 (9)
C7—C8—C9—O1	0.88 (17)	O3—C16—C17—C18	−178.07 (8)
C7—C8—C9—C10	179.45 (10)	C16—C17—C18—C19	173.51 (9)
O1—C9—C10—C15	−173.62 (10)	C17—C18—C19—C20	179.26 (9)
C8—C9—C10—C15	7.77 (15)	C18—C19—C20—C21	177.93 (10)
O1—C9—C10—C11	7.04 (15)		

### Hydrogen-bond geometry (Å, º)

Cg1 is the centroid of the C1–C6 ring.

**Table:**

*D*—H···*A*	*D*—H	H···*A*	*D*···*A*	*D*—H···*A*
O2—H1*O*2···O1^i^	0.94 (2)	1.78 (2)	2.6862 (15)	163.3 (19)
C7—H7*A*···O2^i^	0.93	2.37	3.2526 (18)	158
C16—H16*A*···*Cg*1^ii^	0.97	2.91	3.6117 (16)	130

Symmetry codes: (i) −*x*, −*y*+2, −*z*+2; (ii) −*x*+1, −*y*+2, −*z*+1.
